# Artificial Intelligence and the Implementation Challenge

**DOI:** 10.2196/13659

**Published:** 2019-07-10

**Authors:** James Shaw, Frank Rudzicz, Trevor Jamieson, Avi Goldfarb

**Affiliations:** 1 Women's College Hospital Institute for Health System Solutions and Virtual Care Toronto, ON Canada; 2 Joint Centre for Bioethics University of Toronto Toronto, ON Canada; 3 International Centre for Surgical Safety, Li Ka Shing Knowledge Institute, St Michael’s Hospital Toronto, ON Canada; 4 St Michael's Hospital Toronto, ON Canada; 5 Rotman School of Management, University of Toronto Toronto, ON Canada

**Keywords:** artificial intelligence, machine learning, implementation science, ethics

## Abstract

**Background:**

Applications of artificial intelligence (AI) in health care have garnered much attention in recent years, but the implementation issues posed by AI have not been substantially addressed.

**Objective:**

In this paper, we have focused on machine learning (ML) as a form of AI and have provided a framework for thinking about use cases of ML in health care. We have structured our discussion of challenges in the implementation of ML in comparison with other technologies using the framework of Nonadoption, Abandonment, and Challenges to the Scale-Up, Spread, and Sustainability of Health and Care Technologies (NASSS).

**Methods:**

After providing an overview of AI technology, we describe use cases of ML as falling into the categories of decision support and automation. We suggest these use cases apply to clinical, operational, and epidemiological tasks and that the primary function of ML in health care in the near term will be decision support. We then outline unique implementation issues posed by ML initiatives in the categories addressed by the NASSS framework, specifically including meaningful decision support, explainability, privacy, consent, algorithmic bias, security, scalability, the role of corporations, and the changing nature of health care work.

**Results:**

Ultimately, we suggest that the future of ML in health care remains positive but uncertain, as support from patients, the public, and a wide range of health care stakeholders is necessary to enable its meaningful implementation.

**Conclusions:**

If the implementation science community is to facilitate the adoption of ML in ways that stand to generate widespread benefits, the issues raised in this paper will require substantial attention in the coming years.

## Introduction

Artificial intelligence (AI) has become a topic of central importance to the ways in which health care will change in the coming decades, with recent commentaries addressing potential transformations in clinical care [1,2], public health [3], and health system planning [4]. AI is a general purpose technology (GPT), which means it represents a core set of capabilities that can be leveraged to perform a wide variety of tasks in different contexts of application [5]. Understanding the core capabilities of AI as a GPT, and the ways in which it stands to be incorporated into health care processes, is essential for the implementation research community to contribute to promoting a positive place for AI in the future of health care. We believe that AI has the potential to substantially reconfigure health care, with implications that reach beyond enhancing the efficiency and effectiveness of care delivery. Due to this potential, we suggest that implementation science researchers and practitioners make a commitment to more fully consider the wider range of issues that relate to its implementation, which include health system, social, and economic implications of the deployment of AI in health care settings.

We suggest that the most appropriate language for discussions of AI in health care is actually to discuss machine learning (ML), which is the specific subfield of AI that is currently making the most impact across industries. We then focus on 2 questions about the deployment of ML in health care. First, how should ML be understood in terms of its actual use cases in health care? This question addresses the nature of ML as an *implementation object* [6,7] in health-related contexts. We present a basic framework for thinking about use cases of ML in terms of decision support versus automation and elaborate clinical, operational, and epidemiological categories of these use cases.

Second, what are the unique challenges posed by ML that may require consideration during an implementation initiative? As opposed to focusing on strategies for the adoption of digital technologies in general, which has been addressed extensively in other literature [8-10], we focus on what we understand to be the most important risks arising from the implementation of ML in health care. Our discussion of the risks associated with implementing ML in health care is guided by the work of Greenhalgh et al in the framework for theorizing and evaluating Nonadoption, Abandonment, and Challenges to the Scale-Up, Spread, and Sustainability (NASSS) of health and care technologies [8].

The NASSS framework is based on the premise that when considering influences on whether and how a technology is successfully taken up and used, it is important to keep in mind that “it is not individual factors that make or break a technology implementation effort but the dynamic interaction between them” [8]. The NASSS framework outlines a range of considerations that are relevant to understanding how a technology might be adopted across an entire region or health system, ranging from a focus on the particular health condition in the clinical scenario to the wider political, regulatory, and sociocultural system in which it is to be embedded. In our paper, we examine ML as a GPT that has the potential to apply across clinical conditions and focus our analysis on elements of the NASSS framework: the technology, its value propositions, and the adopters, organizations, and systems into which it might be introduced. We emphasize the evolutionary nature of ML as a GPT and explicitly acknowledge that it will continue to develop and change over the coming years, which is also an important feature of the NASSS framework. We conclude by advocating for further research on the risks posed by ML from an implementation science perspective.

AI has been described in many ways. Using the framing in Agrawal et al, we emphasize that recent advances in AI can be best understood as “prediction technology” [11]. Quite simply, prediction is defined for this purpose as “taking information you have, often called ‘data’, and using it to generate information you don’t have” (PM, p. 24). This newly generated information *estimates* the true information that is missing, leading to the potential for people and technology to take actions that may have otherwise been based on less accurate information.

Predicting illness episodes that might be experienced in the future is an obvious application of AI in this sense, but prediction as we have defined it has many other uses as well. Examples include an automatic translator predicting the phrases of Spanish that correspond to a particular set of phrases in English or a chat bot predicting the most appropriate cluster of words in response to a given query. These examples might not represent the very intuitive understanding of prediction that we have become used to in everyday usage or the way we tend to think of prediction of health-related events and outcomes in health care. However, they represent the prediction of information that we do not have based on information we do have and point toward the potentially widespread applications of AI as a GPT.

The phrase “predictive analytics” is very intuitive with regard to defining AI as a prediction technology, using advanced computer algorithms to predict health-related events from existing data in ways that exceed the ability of individual researchers applying individual analyses [12]. However, AI opens new opportunities for prediction beyond the familiar predictive analytics for hospital admissions, length of stay, and patient survival rates. As a process of filling in missing information, better and cheaper prediction is already being used in new areas, from transcribing audio to enhancing security to informing diagnoses.

At its core, current applications of AI bring statistical modeling, computer code, and advanced computing power to bear on large amounts of representative data. In his recent commentary on the potential of deep learning (a form of AI) to transform health care, Hinton gave the example of deciding whether a patient has a particular disease and explained that a common approach would be to use a simple logistic regression (using data to predict a binary outcome: the patient has the disease or does not). However, he suggested that if there are extremely high numbers of potential influences or predictors of whether the person has the disease, many of which may interact with one another, the prediction challenge becomes much more complex. This is especially the case where we have imperfect knowledge of the causes and correlates of a particular disease. This example also pertains only to binary queries specifically about whether a patient has a single disease, which is different from the typical reasoning processes involved in differential diagnosis among clinicians, where multiple confounding, interdependent outcomes must be considered [13,14].

Specific applications of AI can fall under distinct categories, with AI serving as an *umbrella* concept, covering more specific frameworks. In this paper, we are primarily concerned with the subdomain of AI referred to as ML in which statistical models are automatically (or semiautomatically) induced from data according to some criterion (eg, best expected discriminative power or maximum likelihood given to training data). This means that complex statistical models capable of executing advanced predictions are generated in part by using data to *train* the model to achieve a particular goal.

Often, ML involves *supervised* methods that categorize data points, for example, as images of skin cancer or otherwise given datasets in which all data points (or at least a substantial subset) are associated with a label, ordinal, or category that is meant to be predicted or inferred [15]. This process requires datasets that have the appropriate labels indicating what the data means; in the example of images of skin cancer, each data point would be labeled according to its representation of a mass as *malignant* or *benign* or some variation thereof. Given these labels and the statistical models they help to train, ML can be very effective at determining the category in which any newly available individual data point belongs, thereby being useful in the effort to, for example, identify malignant cancers based on particular images [16].

Much of the power of modern ML also derives from *unsupervised* pattern recognition, in which hidden (or *latent*) aspects of the data are automatically identified by the algorithms and exploited according to the aforementioned criteria. Unsupervised ML can often identify patterns in the data that humans do not even think to look for. Often, these hidden aspects are nonlinear combinations of many parts of the input.

ML can also improve its ability to *take actions* according to these induced hidden patterns and particular functions of cost and reward in a process called *reinforcement learning*. For example, ML can dynamically adapt survey questions to more quickly identify possible diseases [17], dynamically avoid potential communication breakdowns during speech conversation in the assessment of dementia [18], and even recommend treatments directly when using structured institutional data [19]. As so much health care information can be represented digitally, the potential of ML to improve health care practices is profound.

## Methods

### Use Cases of Machine Learning in Health Care

In the remainder of our paper we refer primarily to ML as opposed to AI, focusing our analysis on the concrete possibilities of ML in health care. We can think about use cases of ML in health care in 2 broad ways. The first is through *decision support*, wherein ML algorithms are used to provide some form of input into human decision making. An example is where an algorithm is used to provide more accurate predictions of the outcome of a particular procedure given a particular clinical presentation. This helps to inform a human decision about whether a given procedure is the best course of action. The second is through *automation*, wherein algorithms are used not only to predict an output but also to take action to achieve a particular outcome. An example is the automatic transcribing of a clinical note when dictated into a computer program, resulting in a complete note being added to a patient’s record (technically referred to as Automated Speech Recognition). These 2 broadly defined categories of use cases can be thought of as applying to various types of tasks in health care, and we suggest it is instructive to consider 3 types of tasks as most relevant for the implementation of ML for health: clinical, operational, and epidemiological.

Clinical tasks refer to health-related assessment, intervention, and evaluation, generally performed by qualified health care providers, for example, determining a differential diagnosis. Operational tasks are those related to activities that are ancillary to clinical tasks but necessary or valuable in the delivery of services, such as generating, storing, and retrieving medical records. Finally, epidemiological tasks are those related to more accurately identifying the health needs and outcomes of a set of people within a given population. An example is the development of a warning system for disease outbreak. As epidemiological use cases of ML are related to enhancing the ability of humans to make decisions in the other categories described here (clinical or operational), there are no examples of pure automation for epidemiological tasks that contain an output other than informing a human decision. Hypothetical examples of both decision support and automation are given under each of these categories in Table 1.

This table presents a basic framework for thinking about use cases of ML in health care as falling into 2 primary categories: decision support and automation. These use cases apply in categories of clinical, operational, and epidemiological tasks. As no examples of pure automation exist for epidemiological tasks, no example is presented in that cell.

The considerations most pertinent to the implementation of ML will depend on the particular use case being proposed in a given implementation initiative, and the categories outlined in Table 1 provide a framework for understanding those use cases. The NASSS framework and other work in implementation science for digital health technologies emphasize the importance of attending to the particular value proposition that a new technology offers for health care stakeholders [8,9]. The value proposition of digital technology might be different for different stakeholder groups, and implementation frameworks direct attention to the implications of newly introduced technologies for patients, health care providers, managers, health policymakers, and others [8,25,26]. The clinical, operational, and epidemiological task types presented in Table 1 will correspond to different value propositions for different stakeholder groups, meaning that specific applications of ML might preferentially benefit one group over another, for example, identifying a scheduling process to maximize efficiency in operating costs might preferentially benefit managers over health care providers inconvenienced by a new system. Understanding how value propositions differ for the various stakeholders implicated in a given implementation of ML is an essential consideration for successful adoption and use.

**Table 1:** Examples of use cases in each category of application.

Type of use case	Clinical^a^	Operational^b^	Epidemiological^c^
Decision support	Producing a more accurate prediction of the likely outcome of a particular intervention [20]	Identifying potential staff scheduling changes related to forecasted emergency room volumes [21]	Warning systems for disease outbreak [22]
Automation	Automatically altering insulin treatment in response to monitored glucose-insulin dynamics [23]	Use of robotics for operational tasks in dementia care, such as meal delivery [24]	N/A^d^

^a^Tasks related to the assessment, intervention, and evaluation of health-related issues and procedures, generally performed by qualified health care providers.

^b^Tasks related to activities that are ancillary to clinical tasks but necessary or valuable in the delivery of services.

^c^Tasks related to more accurately identifying the health needs and outcomes of people within a given population.

^d^Not applicable.

The potential value propositions of an ML technology offering decision support versus one offering automation are very different and bring along different sorts of implementation issues. The implementation of decision support systems in health care that do not include applications of ML have been well studied and the difficulties include perceived challenges to autonomy, lack of time, and dissatisfaction with user interfaces [27,28]. Implementation initiatives involving decision support applications of ML will need to consider this past work to develop implementation strategies that more effectively address known challenges.

Implementation initiatives involving automation are likely to face some similar and some different challenges. For example, stakeholder views on the introduction of automated robotics into a variety of health care settings found a widespread lack of interest and understanding and fear of the ways in which work would be disrupted and distributed [29]. Although automation has existed in health care for decades through technologies such as heart rate monitors, the question of how acceptable stakeholders will perceive new forms of automation to be remains an important issue. This point raises the overarching issue of the extent of automation that is possible through applications of ML, linked to speculation about whether ML will mostly *augment* or actually *replace* health care providers’ work [1,30].

### Augmentation and Replacement of Health Care Work

We agree with a growing chorus of health care providers and researchers who suggest that ML will primarily serve to *augment* as opposed to *replace* the work of humans in the provision of health care in the near term [31], despite applications of automation in health care. This is because the role of ML in the current generation of capabilities functions at the level of the *task*, and not at the level of an entire *job*. Agrawal et al explained that “the actual implementation of AI is through the development of tools. The unit of AI tool design is not ‘the job’ or ‘the occupation’ or the ‘the strategy’, but rather ‘the task’.” (p. 125). Therefore, for a health care provider to be entirely replaced, every single task performed by that provider would need to be automated by an ML tool or handed off to a different human.

The complete automation of the full range of human tasks involved in providing clinical care is not yet possible; activities such as making treatment decisions based on a differential diagnosis that integrates data from laboratory investigation, visual observation, and patient history are still too complex for automation. In emphasizing this point, we are suggesting that although much of the hype about AI (and specifically ML) in health care has focused on its potential role in *automating* processes of health service delivery, it is more likely that near-term applications of ML will fall under the category of *decision support*.

Further comments about prediction tasks and decision tasks will help to clarify this point. As stated earlier, ML applications fundamentally perform some form of prediction. The specific instance of prediction that the application is performing may be thought of as the *prediction task*, which may be paired with a complementary *decision task*. The decision task is where the newly generated information is used to select a particular action in a given context. In applications of ML that function as decision support, the decision task is performed by a human. As ML diffuses, an important new challenge for health care providers is to make choices using the predictions that arise from decision support applications of ML, involving new forms of input to clinical thought processes related to risks, benefits, and previously unrecognized influences on health. The examples of decision support in Table 1 involve generating better information to inform human decision making.

In applications of ML that function as automation, both the prediction task and the decision task are accomplished by machines. A clear example is self-driving cars. The sensors surrounding the car enable predictions of the best direction in which the car should travel. However, it is the *selection* from a predetermined set of actions and *execution* of one action over another that makes self-driving cars an example of automation as distinct from one of decision support. ML is not yet sophisticated enough to complete these selection and execution functions for many health care tasks, across both clinical and operational levels.

As prediction tasks become more amenable to being performed by ML, decision tasks become more valuable [5,32]. This is because predictions are improved, meaning that decisions can be made with greater confidence and impact. The enhanced value of these decisions represents the potential value of ML as a decision support tool and illustrates the potential breadth of value propositions that could arise from this technology with a wide range of implications for the implementation process. However, for decision support to be valuable in health care, the outputs of algorithms must have a clear entryway into the human decision-making processes that pervade health service delivery. This points us toward one of a series of important issues raised from an implementation science perspective on the introduction of ML in health care settings, which we turn to next.

## Results

### Unique Considerations for Implementation Science

We have described use cases (and attendant value propositions) of ML in health care as more likely relating to decision support and less likely to automation, which begins to illustrate the implementation object of focus in ML initiatives [6,7]. In many cases of decision support, the implementation object is actually not all that different from the statistical tools that are already used as part of common practice, such as risk prediction. In cases of automation, there are similarly many examples of technologies that have already been successfully implemented in health care settings (such as automatic transcription mentioned earlier). However, ML as a GPT raises a number of issues that run across use cases and might be anticipated as unique in comparison with implementation projects for other digital technologies.

Best practices of implementation for digital innovations [8,9,33] will be fundamental to the adoption of ML in health care. Here, we discuss considerations that might appear in implementation projects involving ML that may be less likely to appear in implementation projects involving other digital technologies and yet stand to have a potentially strong influence on the success of such projects. We organize this section based on distinct levels of consideration that are presented in the NASSS framework that we have not yet addressed [8,26]: health care providers, patients and the public, health care organizations, and health policy and systems. Although we consider the primary considerations of health technology vendors working on the development of ML application in health care to be outside the scope of this paper, we acknowledge this is a gap in the literature that requires attention.

### Health Care Providers

Health care providers are those responsible for doing the actual work of health care delivery and are being increasingly expected to adopt and use new technologies in health care environments. We suggest that the core considerations or risks of the implementation of ML for health care providers will fall into the categories of meaningful decision support and explainability.

#### Meaningful Decision Support

For ML to function as decision support in a way that is valuable to health care stakeholders, the outputs of algorithms must have a meaningful entryway into decision making. From an operational or epidemiological perspective, isolated analyses of risk prediction may help to inform resource allocation and subsequent analysis decisions fairly simply. However, from a clinical perspective, algorithms that perform isolated risk prediction may be less useful. Clinical decision making is a complex process involving the integration of a variety of data sources, incorporating both tacit and explicit modes of intelligence [34-36]. To inform this decision-making process more intuitively, attention is increasingly being devoted to communication tools such as data visualization [37]. The nature and value of these communication tools are central to the implementation process, helping to determine whether and how algorithmic outputs are incorporated in everyday routine practices. This point primarily relates to the decision support use case across clinical, operational, and epidemiological tasks.

#### Explainability

There is a growing concern in the AI community related to the explainability of the results achieved by ML algorithms, wherein the ways in which algorithms enhance the performance of prediction can often not be understood [38]. As a result of the processes described earlier in this paper, the ways in which data are being used to train algorithms cannot be traced out in sequential, logical detail. Hence, the actual ways in which models achieve their results are in some instances not knowable even to the computer scientists who create them. Evidence-based medicine rests on a foundation of the highest standards of explainability; medical decision making aspires to incorporate a sound understanding of the mechanisms by which diseases and their treatments function and the particular treatments that have demonstrated the greatest benefits under particular experimental circumstances (in addition to patient needs and values [35,39,40]). The lack of understanding of those mechanisms and circumstances poses challenges to the acceptability of ML to health care stakeholders. Although the issue of explainability relates clearly to decision support uses cases of ML as explained here, the issue may apply even more profoundly to automation-focused use cases as they gain prominence in health care.

### Patients and the Public

The issues of public trust and public input into the governance of ML initiatives in health care have been widely discussed as the popularity of AI has grown, with advocates suggesting that future developments of AI ought to be explicitly supporting a broader public interest. We suggest that 2 pairs of issues frame the risks of ML related to patients and the public. The first pair is privacy and consent and the second is representative data and algorithmic bias.

#### Privacy and Consent

The training of ML models requires large amounts of data, which means that applications of ML in health will likely rely on health-related data from patients and the public. As governments and other actors internationally become interested in developing applications of ML, health-related data are increasingly made available to private entities with the capability of producing AI applications that are relevant to peoples’ health [41-43]. Currently, data from wearable devices such as smart watches and mobile apps are not widely covered by health information legislation [44], and many health-related apps have unclear consenting processes related to the flow of data generated through their use [45]. Furthermore, data that are de-identified may be reidentifiable when linked with other datasets [46]. These considerations create major risks for initiatives that seek to make health data available for use in the development of ML applications, potentially leading to substantial resistance from health care providers such as that seen in primary care in Denmark in recent years [42]. This will be particularly important for population and public health use cases that require data from very large segments of the population. The meaning of consent and strategies to maintain patient privacy are central considerations to ML implementation initiatives. The related issues of privacy and consent pertain especially to clinical and epidemiological use cases of ML in both decision support and automation categories, as data from patients /or the public are essential to train algorithms in these areas (whereas operational use cases may only rely on other forms of data, such as clinical scheduling histories).

#### Representative Data and Algorithmic Bias

Algorithms are only as good as the data used to train them. In cases where training data are partial or incomplete or only reflect a subset of a given population, the resulting model will only be relevant to the population of people represented in the dataset [47]. This raises the question about data provenance [30,48] and represents a set of issues related to the biases that are built into algorithms used to inform decision making. One high profile example was the hiring bias exhibited when algorithms were used to make hiring decisions at Amazon, resulting in only men being advanced to subsequent stages of hiring [49]. This is notable in part because the algorithm performed extremely well based on the available data, simply extending the bias that already existed in hiring practices at the company. When applied to health care of public health, data provenance and potential bias in training data represent important issues that are likely to be of major concern for the stakeholders involved in the implementation of an ML initiative. Public health has health equity as a primary goal, and representativeness in terms of which populations can be addressed by an ML initiative will be a central consideration.

A further challenge with the nature of the data on which algorithms are trained relates to *concept drift*, a phenomenon where data on which an algorithm is trained change over time (or become out of date), which changes the performance of the algorithm as new data are acquired [50]. The possibility of concept drift means that those overseeing the performance of ML-based technologies in health care must identify strategies to determine how well the algorithm deals with new data and whether concept drift is occurring. Applications to support this effort are emerging in the literature [51].

The issues addressed here apply most clearly to ML applications that use patient data to inform clinical and epidemiological use cases that enhance clinical care and health system planning. And although the use of public data will likely be the most contentious issue in this domain, the challenges of representativeness and bias apply to all ML use cases across decision support and automation domains.

### Health Care Organizations

Health care and public health systems are composed of independent organizations that need to develop and execute strategies within the limits of the resources available to them. Organizations have been the driving force behind the adoption of many innovations in health care and have a collection of considerations that are unique from the broader systems of which they are a part. We suggest that the issue of security and computational resources become particularly important for organizations as they adopt ML initiatives in health care and public health.

#### Security

As data are collated and stored for training ML models, the risk and potential severity of security breaches grows. The global attack of health care organizations using *WannaCry* ransomware in May 2017 shows the vulnerabilities of even well protected health data to malicious interests. This particular attack is estimated to have affected 200,000 systems in over 150 countries, indicating the potential scope of security problems as the value of data grows [52]. Strategies to prevent such security breaches on Web accessible health data are now being proposed in the literature [53,54], and the high profile of security issues makes this a particularly important issue as ML applications develop in health care and public health. The issue of security transcends any particular use case of ML and includes any applications or analysis that relies on big data more generally.

#### Computational Resources

Advanced applications of ML require substantial computing power, with some predictive analyses and training models requiring up to several weeks to run. The more extensive the computing support, the more efficient ML applications will become, raising the question of the cost and availability of such advanced computing power for health care organizations. Health care is publicly funded in many countries around the world, and public support to secure the resources to fund the necessary computing power may not be present. Cloud-based analytics present an opportunity and a challenge for health-related organizations in relation to the issue of computational resources. Cloud-based data analysis means that organizations would not need to own computational resources directly [55] but also introduces the potential challenges of data safety. These issues are relevant to the training phase of a newly developed algorithm, but of course, less computing power is required to simply apply algorithms that have been generated and trained elsewhere. How data are stored and processed is thus also an important consideration in ML implementation initiatives. The issue of computational resources also applies more generally than any given ML use case, related to the development and functioning of many kinds of AI algorithms.

### Health Policy and Systems

The challenges associated with ML initiatives at the level of health policy and systems are extensive. These include broad legislative frameworks related to emerging health-related technologies more generally [56] and to the innovation procurement systems that vary across health system settings [57,58]. The policy issues presented by ML in health care are beginning to garner more attention [42,43], but here we present one issue that we have not seen addressed in health care or public health literature: the challenge of scalability.

#### Scalability and Normal Accidents

A major challenge that extends beyond any single implementation of ML, and therefore requires a system-wide view, relates to the *scalability* of ML. Scalability in this sense refers to the unanticipated effects of the appearance of multiple ML technologies that will inevitably interact with one another by some means. As applications of ML proliferate across health care and public health, eventually some algorithmic outputs will confront others. The effects of this interaction are impossible to predict in advance, in part because the particular technologies that will interact are unclear and likely not yet implemented in the course of usual care.

Health care represents what Charles Perrow referred to as a complex system or a system in which processes are tightly linked to one another and interact in unintended ways in the effort to achieve the goals of the system [59]. This acknowledgement has led to the high reliability movement in health care and other industries [60], intending to implement management strategies that could mitigate against the risk of disasters arising from such immense complexity. Perrow’s work was titled *Normal Accidents: Living with High Risk Technologies*, suggesting that in systems characterized by complexity and the use of advanced technologies, accidents are bound to happen [59]. This basic point about the seeming inevitability of accidents in the context of complex systems and new technologies underscores the significance of the scalability challenge of ML in health care. We suggest that implementation scientists will need to consider the unintended consequences of the implementation and scale of ML in health care, creating even more complexity and greater opportunity for risks to the safety of patients, health care providers, and the general public. ML safety will likely need to become a dedicated focus of patient safety research internationally. This point about scalability frames the broader challenge for implementation scientists who are committed to a system-wide perspective on health innovations and relates not only to each type of use case identified in our framework but also to the interactions between them as well.

## Discussion

### Intersecting Issues in the Future of Health Care

In our brief Discussion section, we outline 2 overarching issues that we consider to frame the challenges facing health care systems that are hoping to adopt ML in the coming years. The discussion here is informed by the explicit recognition in the NASSS framework that both the technology and context in which innovations are being introduced shift and change over time. Greenhalgh et al suggest that although the levels of the framework can be distinguished analytically, “at an empirical level they are inextricably interlinked and dynamically evolving, often against a rapidly shifting policy context or continued evolution of the technology” (p. 14). Our assessment of the 2 issues we address here is intended to represent the connections between the changes that will be required as the policy context and technology evolve concurrently. The first is the issue of the role of corporations in health-related applications of ML, and the second is the issue of the role of ML in the evolving nature of health care.

### The Role of Corporations

As the innovations enabled by ML have taken on a more powerful role in driving global economies, corporations have strategically sought to acquire larger amounts of more diverse data to boost their capacity to develop ML algorithms [61]. The shifting focus of many large corporations to the collection and manipulation of data characterizes what Zuboff refers to as *surveillance capitalism*, a relatively recent phenomenon in the global economy that relies on data for innovation and corporate success. The more that large corporations enter the health care industry with the power to collect, store, and use data, the more intertwined health care will become with the corporate realities of these large, multinational companies [62].

As large corporations acquire more data and develop more sophisticated forms of ML that transcend any individual geographical region, the implications for domestic health care policy are at risk of being overlooked. Although recent efforts to create regional protections around data collection and use have appeared to make an impact, such as the General Data Protection Regulation in Europe, health care policy is well behind. In cases where health-related data are already being stored in a country other than where the user is living, what are the regulations on how those data can be used? Where users voluntarily engage with technologies that collect their data for explicit health-related use by a corporation outside of their political jurisdiction, what legislative frameworks apply to protect patients and the public? These issues represent the important challenge of making health policy matter when conventional political boundaries are less able to contain the potential of large corporations to develop and use their technological capabilities.

### The Changing Nature of Artificial Intelligence–Enabled Health Care

AI applications represent a potential impetus for major change in the institutions that constitute health care. In this sense, the term institution refers not just to the organizations in which health care providers work but to a complex collection of cognitive, cultural, regulative, and moral influences that shape the way that health care workers see their work and their lives [63]. The social sciences have worked to provide clear definitions of institutions through decades of research and theory [63-65]. Scott explained that institutions are combinations of 3 pillars: norms of *the way things are usually done around here* (cultural-cognitive influences), laws and regulations (regulative influences), and assumed moral codes (normative influences) [63]. Health care represents a confluence of institutions understood in this sense, many of which are naturally oriented toward maintaining some version of the status quo. Particularly for members of institutions who maintain power over resources, such as the medical profession, embracing institutional change is a point of resistance and difficulty.

We suggest that ML will confront the realities of entrenched institutions through issues such as meaningful decision support and explainability described earlier. These 2 issues represent the authority of health care providers over the decisions that come to define health care as a multi-institutional field, both in terms of their rightful positions within the system and the fabric of decision making that has always defined health care processes. These issues point toward an important challenge that we suggest implementation scientists must grapple with: the changing nature of health care work. In *Prediction Machines*, the authors explain that as AI technology develops, “the value of substitutes to prediction machines, namely human prediction, will decline. However, the value of complements, such as the human skills associated with data collection, judgment, and actions, will become more valuable.” (p. 81). As the implementation science community considers how to encourage the adoption of ML technologies, it will also need to consider how such technologies stand to change the ways in which health care planning, decision making, and delivery are understood and the evolving role of human health care providers within that context.

The challenges described here refer to unique considerations of ML that pose novel challenges to implementation beyond the work of promoting the routine use of technologies among health care providers. We suggest that the hype and high stakes of ML make these issues more prominent in the mindsets of health care stakeholders and therefore more likely to impact upon an ML implementation project. The implementation science community will need to establish strategies to address these issues as ML becomes more prominent, each of which requires ongoing work to be adequately addressed.

### Conclusions

In this paper, we have provided an overview of ML for implementation scientists informed by the NASSS framework, outlining the use cases of ML as falling into the categories of decision support and automation. We suggest these use cases apply to clinical, operational, and epidemiological tasks and that the primary ways in which ML will enter into health care in the near term will be through decision support. We then outlined unique implementation issues posed by ML initiatives from 4 perspectives, those of health care providers, patients and the public, health care organizations, and health policy and systems.

Ultimately, we suggest that the future of ML in health care remains positive but uncertain, as support from patients, the public, and a wide range of health care stakeholders is necessary to enable its meaningful implementation. However, as applications of ML become more sophisticated and investment in communications strategies such as data visualization grows, ML is likely to become more user-friendly and more effective. If the implementation science community is to facilitate the adoption of ML in ways that stand to benefit all, the issues raised in this paper will require substantial attention in the coming years.

## Abbreviations

AI: artificial intelligence
GPT: general purpose technology
ML: machine learning
NASSS: Nonadoption, Abandonment, and Challenges to the Scale-Up, Spread, and Sustainability
